# Supplementary material for: Patterns of Intron Gain and Loss in Fungi
Source: PLoS Biol. 2004 Nov 30;2(12):e422. doi: 10.1371/journal.pbio.0020422 (PMC532390; doi:10.1371/journal.pbio.0020422)
Supplement: Table S1 — Also available at http://genes.mit.edu/NielsenEtAl/. (4.3 MB ZIP). [file pbio.0020422.st001.zip › NielsenEtAl/html/1343.html]

AN0583.1.NCU07712.1.MG04179.1.FG09766.1


```
 CLUSTAL W (1.82) Multiple Sequence Alignments - Introns Inserted


Sequence 1: MG04179.1	880 aa
Sequence 2: FG09766.1	897 aa
Sequence 3: NCU07712.1	934 aa
Sequence 4: AN0583.1	936 aa
Alignment Length: 953 aa
Number Identitical Residues: 402 aa
Alignment Score (without introns) 20100


MG04179.1 	MPRRAASPAASEHEIDILGSIFANDNDTEVKAKAKKRTGG---HDEIDLDIDALLNGAED
NCU07712.1	MPRRAASPTPSENEVDIAGALFNNDSDFEDNSSKHHTKKGAVTNSGLDLDFNDLLGNGDD
FG09766.1 	MPRRGASPTPSEGEIDIFGSLYPGETD--------NANGG---NGGDDFDFDGLLNNPEP
AN0583.1  	MPSRAASPALSENEFDITGALFQNDSDSDAETQKPTKRKKVPAAPNVNLDFLGEANAGDS
          	** *.***: ** *.** *::: .:.* . .:.         :    ::*:    .  : 

MG04179.1 	------GGDGDDEALIALQQAASFRKTTNLKGKTGKKSGGFQAMG1LNPSLLQAITRKGF
NCU07712.1	GLPSFNGEGDDDEAFIASLTRSSQRKSSNIQGKSVKKGGGFQAMG1LNAHLLRAITRKGF
FG09766.1 	------GNDDTDEAFIALQQAASFRKATNLKGRTVKKGGGFQAMG1LNNNLLKAITRKGF
AN0583.1  	--------DEDDEAFIAEKQTSANRKSANLKGRTVKKGGGFQAMG~LNANLLKAIARKGF
          	        .  ***:**    :: **::*::*:: **.******* **  **:**:****

MG04179.1 	AVPTPIQRKSIPLILDRRDVVGMARTGSGKTAAFVIPMIERLRAHSARVGARALIMSPSR
NCU07712.1	SVPTPIQRKAIPLILERKDVVGMARTGSGKTAAFVIPMIERLKGHSPRVGSRALIMSPSR
FG09766.1 	SVPTPIQRKAIPLILDRKDLVGMARTGSGKTAAFVIPMIEKLRAHSARFGTRALIMSPSR
AN0583.1  	SVPTPIQRKTIPVIMEDQDVVGMARTGSGKTAAFVIPMIEKLKSHSTKFGARGLILSPSR
          	:********:**:*:: :*:********************:*:.**.:.*:*.**:****

MG04179.1 	ELALQTLKVVKEFGKGTDLKTVLLVGGDSLEDQFGFMTTNPDIIIATPGRFLHLKVEMSL
NCU07712.1	ELALQTLKVVKELGRGTDLKTVLLVGGDSLEEQFGMMASNPDIIIATPGRFLHLKVEMNL
FG09766.1 	ELAIQTLKVVKEFSRGTDLKCVLLVGGDSLEEQFGYMAANPDIVIATPGRFLHLKVEMSL
AN0583.1  	ELALQTLKVVKELGKGTDLKSVLLVGGDSLEEQFGMMAGNPDIVIATPGRFLHLKVEMNL
          	***:********:.:***** **********:*** *: ****:**************.*

MG04179.1 	DLSSIKYVVFDEADRLFEMGFATQLTEILHSLPPSRQTLLFSATLPRSLVEFARAGLQDP
NCU07712.1	SLASIKYVVFDEADRLFEMGFATELTEILHALPPSRQTLLFSATLPSSLVEFARAGLQEP
FG09766.1 	DLSSIKYVVFDEADRLFEMGFAAQLTEILHALPPSRQSLLFSATLPASLVEFARAGLQDP
AN0583.1  	DLSSIKYVVFDEADRLFEMGFAAQLTEILHGLPSTRQTLLFSATLPKSLVEFARAGLQDP
          	.*:*******************::******.**.:**:******** ***********:*

MG04179.1 	SL0----------------------------------DVIKMPTGTPEG---FKEDKDEG
NCU07712.1	SL~VRLDAETKVSPDLESAFFAVKGGEKEGALLHLLHDVIKVPLGPPEG---TKEESDEL
FG09766.1 	SL~VRLDAEQKVSPDLESAFFAVKGAEKEGSLLHILHDVIKMPVGSPEG---VNNDSEKG
AN0583.1  	TL~VRLDTESKISPDLQNAFFSVKSAEKEGALLYILNEVIKMPTGPTEASLRLKEKGPED
          	:*    .:. . :.. ..:  : .......:      :***:* *..*.:   ::.  : 

MG04179.1 	SKKRKR----GPDRPNAKEKPTEHSTIIFTATKFHVEYLTSILVQAGYAVSHAYGALDQT
NCU07712.1	QARKRK----REYRPNPKEKPTEYSTIIFTATKHHVEYIANLLKLAGFAVSYVYGSLDQT
FG09766.1 	SKKRKR----GAD--GGSGKPTEHSTIIFTATKHHVEYLANLLIYAGFAVSYVYGSLDQT
AN0583.1  	SKNKKRKRAEMERAVNMKESPTKHSTIVFAATKHHVDYLYSLLREAGFAVSYVYGSLDQT
          	. .:::. :.     . . .**::***:*:***.**:*: .:*  **:***:.**:****

MG04179.1 	ARKIQVEDFRRGKTNILVVTDVAARGIDIPVLANVINYDFCDQPKVFVHRVGRTARAGQK
NCU07712.1	ARLIQVDNFRRGRTHILVVTDVAARGIDMPALANVINYDFPSQPKIFVHRVGRTARAGQR
FG09766.1 	ARRIQVEDFRMGKTNILVVTDVAARGIDIPVLANVINFDFPPQPKVFVHRVGRTARAGQR
AN0583.1  	ARKIQVQNFRTGISNILVVTDVAARGIDIPILANVINYDFPSQPKIFVHRVGRTARAGRK
          	** ***::** * ::*************:* ******:**  ***:************::

MG04179.1 	GWSYSLVSDIDAPYLLDLQLFLGRRLVVGQDTSAGANFASDVVLGALQRNSIETNVEWVE
NCU07712.1	GWAYGLVRQSDVPYLLDLQLFLGRKLIIGHDQKN-PSFAADVVVGTLKRDGVDVNIEWVE
FG09766.1 	GWSYSLVRDTDAPYLIDLQLFLGKKLVVGQETKN-PSFSEDVVVGALKRDPVEGHVEWFN
AN0583.1  	GWSYSLVRDADAPYLLDLQLFLGRKLVLGRESDQ-VNFAEDVAVGSLPRDGLSQTCEWVS
          	**:*.** : *.***:*******::*::*:: .   .*: **.:*:* *: :.   **..

MG04179.1 	KVVQESHDIALMRSVVVKAQKQYLRTRVSASSQSAKRARELTASRAWSQPHLIFGINTDD
NCU07712.1	KALKESADLKALKGVAAKAEKLYMKTRNSASSQSAKRAREVTQSRGWTQLHPLFGEEAAE
FG09766.1 	KSLHESEDINALRGVAVKAEKLYLRTRNSAASQSAKRSKELVGSQGWTQLHPLFGEDVDG
AN0583.1  	RVLDDDADIFAQRAVSTKGEKLYLRTRNAASAESAKRAKQVVTSDNWTAVHPLFQDEASN
          	: :.:. *:   :.* .*.:* *::** :*:::****::::. *  *:  * :*  :.  

MG04179.1 	TEALRVEMLAKISGFKPQETVFEIGHG--GKGTIS----EAVEVMKQLRKRAPVRK---S
NCU07712.1	AQAARDDLLSRINRFKPQETIFELGPK--GKSSRN----KAAEVVRNMRSRFKERKTTND
FG09766.1 	AEQARVDMLARISGFRPQETIFEIGGR--RDKGTT----EAAEVMKQLRKRITPRRQTEA
AN0583.1  	LEAEREKMLARIGGYRPQETIFEVQNRRGGKGGKASEPDEALDSIKRVRSTLEAKKKQRA
          	 :  * .:*::*. ::****:**:    . .    :...:* : ::.:*.    ::    

MG04179.1 	KTDKDADDEDED---------VPVIKRAKSDESSDEDASFDEDDFVAVNDDSDEELEVTV
NCU07712.1	EDDEDVDMEDAEGKPDGEETNAFEDFEDEEEEGEAEEAEEAEAKEDPYADDSDSEMEVTV
FG09766.1 	KADDDFEGIGDD---------IPAAAEAGAD-SDEEEVVMDEMD---VDEESDDGLEVTV
AN0583.1  	QAEELEATTNTDG--AEVDGDAFSDLEGDNANIPDNMSLASESDLEVTFSSYNTTDKASK
          	: :.     . :.     . .     .    .   :     * .     .. :   :.: 

MG04179.1 	SNNADSAK--NASAWRDSEHFMTYTPRQSNVAEERGYGVNAGSNG-ANFLEAARDVAMDI
NCU07712.1	SSSMHTKKKGGPVNFQDPEIFMSYTPRTTSLAEEKAYGVHSGGYSGNSFVEAARDATMDL
FG09766.1 	TN-TNSKK--GRTDWRDSEVFMSYTPRTFNAAEERGYGVSSGGQDPSNFVEAARGVTMDL
AN0583.1  	SNSNSKSDSTPTTLFQNPEYFMSYTPANTSLAEDRAYGVHSGTNA--NFASATRNATMDL
          	:..  . ..     :::.* **:***   . **::.*** :*     .* .*:*..:**:

MG04179.1 	ANDEKSTSFGAPTRTTMRWDKKNAKYVSRAHDEDGSRGNTKMIRGESGVKIAASFKSGRF
NCU07712.1	TNDESAKNFGLPTKSKMRWDKRHSKYVAVANDEDGSKG-AKMIRGESGVKIAASFKSGRF
FG09766.1 	TNDENAKSFGEPTRSKMRWDKKSRKYVSRENDEDGSKG-AKMIRGESGVKIAASFQSGRF
AN0583.1  	QADEGGKGFGEP-RTLMRWDKRHKKYVSRQNDEDGSKG-TKLVRGESGAKIAATFRSGRF
          	  ** ...** * :: *****:  ***:  :*****:* :*::*****.****:*:****

MG04179.1 	DRWRKDNRLGKLPGVGEAETG-LPRGMGGGGFGGGGRRFNHKREDAPKEADKFRDDYHVR
NCU07712.1	DRWRKDNRLGKLPTIGETEK---SQLIRNFGAQPGQPRYKHKMEKAPKDADKFRDDYHVR
FG09766.1 	DKWKRANRLGKLPHVGEQVR---PGSASHAANLPSGVRYKHKQERAPKEADKYRDDFEVR
AN0583.1  	DAWKKGKRVGRLPRVGEAETPGLAADLGGSGGSFGGKRFRHKSEKAPKAADPLRGDYEKM
          	* *:: :*:*:** :**    . .      .   .  *:.** * *** **  *.*:.  

MG04179.1 	KKRVAEAKEKRIGKFRDGE-GSKREIKNNDDIRKARKIKELKMRKNARPARKKKN
NCU07712.1	KKRVAEAKEKRIGKYKDGE-GSKRELKTATDIRKARAVAEKKREKNARPAKRQKR
FG09766.1 	KKRVDEAREKRVGRFRDGM-GSKKELKGREDIRKARIEKEKKRLKNARPTRRK--
AN0583.1  	KKKAEAARERAASKVGGVTSGGKSEIRNTDDIRKARKLKQKRREKNARPSRKK--
          	**:.  *:*:  .:  .  :*.* *::   ******   : :  *****::::
```
